# Supplementary material for: Multimodal biomarker based on temporal complexity of eye movements and pupil diameter in attention-deficit/hyperactivity disorder
Source: PLOS Ment Health. 2025 Oct 9;2(10):e0000456. doi: 10.1371/journal.pmen.0000456 (PMC12798525; doi:10.1371/journal.pmen.0000456)
Supplement: S1 Table — (PDF) [file pmen.0000456.s001.pdf]

S1 Table. Correlation matrix of predictive features.

	Pupil Size	Hor FuzzyEn	Vert FuzzyEn
TD vs ADHD			
Pupil Size	-	$r = -0.116$ ($p = 0.500$)	$r = -0.211$ ($p = 0.216$)
Hor FuzzyEn	-	-	$\mathbf{r = 0.751}$ ($\mathbf{p < 0.001}$)
Vert FuzzyEn	-	-	-
TD vs drug-naïve ADHD			
Pupil Size	-	$r = -0.166$ ($p = 0.373$)	$r = -0.208$ ($p = 0.263$)
Hor FuzzyEn	-	-	$\mathbf{r = 0.767}$ ($\mathbf{p < 0.001}$)
Vert FuzzyEn	-	-	-

Pearson's correlation coefficients (r) among the three predictive features for the TD vs ADHD and TD vs drug-naïve ADHD classification tasks. Values meeting the criteria for potential multicollinearity ($|r| > 0.7$) are highlighted in bold text.
