# Supplementary material for: Multimodal biomarker based on temporal complexity of eye movements and pupil diameter in attention-deficit/hyperactivity disorder
Source: PLOS Ment Health. 2025 Oct 9;2(10):e0000456. doi: 10.1371/journal.pmen.0000456 (PMC12798525; doi:10.1371/journal.pmen.0000456)
Supplement: S2 Table — (PDF) [file pmen.0000456.s002.pdf]

S2 Table. Performance of the Lasso logistic regression model across all feature combinations.

Model Features	TD vs ADHD		TD vs drug-naïve ADHD	
	AUC-ROC	AUC-PR	AUC-ROC	AUC-PR
Pupil Size	0.76	0.65	0.77	0.65
Hor FuzzyEn	0.65	0.59	0.78	0.60
Vert FuzzyEn	0.72	0.71	0.75	0.67
Pupil Size + Hor FuzzyEn	0.79	0.73	**0.83**	0.68
Pupil Size + Vert FuzzyEn	**0.83**	**0.79**	0.82	**0.73**
Hor FuzzyEn + Vert FuzzyEn	0.72	0.70	0.75	0.57
Pupil Size + Hor FuzzyEn + Vert FuzzyEn	0.79	0.73	0.78	0.63

Comparison of performance for the Lasso logistic regression model across all feature combinations for the TD vs ADHD and TD vs drug-naïve ADHD classification tasks. The highest value in each column is highlighted in bold text. (TD, typical development; ADHD, attention-deficit/hyperactivity disorder; AUC-ROC, Area Under the Receiver Operating Characteristic Curve; AUC-PR, Area Under the Precision-Recall Curve)
