# Supplementary material for: Multimodal biomarker based on temporal complexity of eye movements and pupil diameter in attention-deficit/hyperactivity disorder
Source: PLOS Ment Health. 2025 Oct 9;2(10):e0000456. doi: 10.1371/journal.pmen.0000456 (PMC12798525; doi:10.1371/journal.pmen.0000456)
Supplement: S3 Table — (PDF) [file pmen.0000456.s003.pdf]

S3 Table. Performance comparison across different classifiers.

Model Features	Lasso Logistic		Standard Logistic		Firth's Logistic	
	AUC-ROC	AUC-PR	AUC-ROC	AUC-PR	AUC-ROC	AUC-PR
TD vs ADHD						
Pupil Size	0.76	0.65	0.76	0.64	0.76	0.64
Hor FuzzyEn	0.65	0.59	0.65	0.59	0.65	0.59
Vert FuzzyEn	0.72	0.71	0.69	0.68	0.69	0.68
Pupil Size + Hor FuzzyEn	0.79	0.73	0.77	0.68	0.77	0.68
Pupil Size + Vert FuzzyEn	**0.83**	**0.79**	**0.80**	**0.71**	**0.80**	**0.72**
Hor FuzzyEn + Vert FuzzyEn	0.72	0.70	0.64	0.61	0.65	0.63
Pupil Size + Hor FuzzyEn + Vert FuzzyEn	0.79	0.73	0.77	0.69	0.77	0.71
TD vs drug-naïve ADHD						
Pupil Size	0.77	0.65	0.75	0.57	0.75	0.57
Hor FuzzyEn	0.78	0.60	0.79	0.59	0.79	0.59
Vert FuzzyEn	0.75	0.67	0.75	0.66	0.75	0.66
Pupil Size + Hor FuzzyEn	**0.83**	0.68	**0.82**	**0.67**	**0.83**	**0.68**
Pupil Size + Vert FuzzyEn	0.82	**0.73**	0.79	0.64	0.80	0.65
Hor FuzzyEn + Vert FuzzyEn	0.75	0.57	0.74	0.59	0.75	0.57
Pupil Size + Hor FuzzyEn + Vert FuzzyEn	0.78	0.63	0.79	0.64	0.80	0.66

Comparison of model performance across all feature combinations and three different logistic regression (Lasso Logistic, Standard Logistic, Firth's Logistic) classifiers, separated by classification task. The highest value in each column for each task is highlighted in bold text. (TD, typical development; ADHD, attention-deficit/hyperactivity disorder; AUC-ROC, Area Under the Receiver Operating Characteristic Curve; AUC-PR, Area Under the Precision-Recall Curve)
