# Supplementary material for: Multimodal biomarker based on temporal complexity of eye movements and pupil diameter in attention-deficit/hyperactivity disorder
Source: PLOS Ment Health. 2025 Oct 9;2(10):e0000456. doi: 10.1371/journal.pmen.0000456 (PMC12798525; doi:10.1371/journal.pmen.0000456)
Supplement: S1 Text — (PDF) [file pmen.0000456.s004.pdf]

S1 Text. Analysis of Gaze Distribution

To evaluate the spatial distribution of gaze points during the fixation task, we generated heatmaps for each participant group (TD, ADHD, and drug-naïve ADHD) using raw, unfiltered horizontal and vertical gaze coordinate data. Furthermore, to quantify the dispersion of gaze, we calculated the standard deviation of the horizontal (SDx) and vertical (SDy) gaze positions for each participant. Group differences were evaluated using the non-parametric Mann-Whitney U test, as the assumption of normality for the data was not met. The resulting heatmaps (S1A Fig) show that for all groups, gaze is concentrated on the central fixation cross; however, a larger dispersion of gaze was visually confirmed in the ADHD and drug-naïve ADHD groups compared to the TD group. This finding was also supported by statistical analysis, which revealed significantly larger standard deviations in both horizontal and vertical gaze positions for the ADHD groups compared to the TD group (S1A Table, all $p < 0.05$).

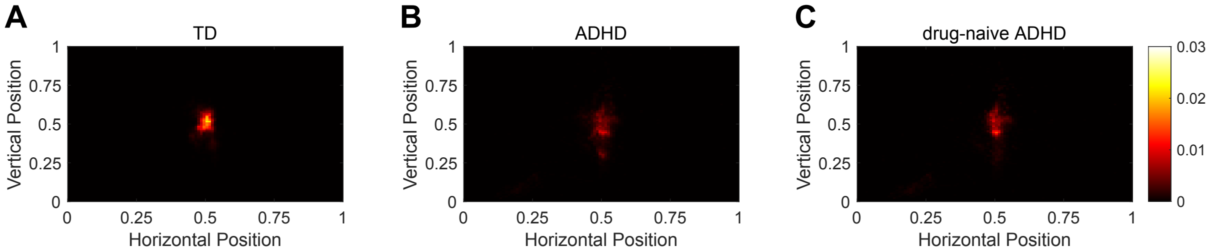

S1A Fig. Gaze distribution during the fixation task. Heatmaps of gaze distribution during the fixation task for the (A) TD, (B) ADHD, and (C) drug-naïve ADHD groups. The coordinates of each heatmap are normalized to a range of 0 to 1 based on the display dimensions, and the aspect ratio of the figure is identical to that of the display. The color scale represents the density of gaze points, with warmer colors indicating higher density.

S1A Table. Statistical comparison of gaze position standard deviation.

Feature	Group	Mean ± SD	*p*-value (vs TD)
SDx	TD	0.016 ± 0.012	-
	ADHD	0.049 ± 0.055	**0.019**
	drug-naïve ADHD	0.050 ± 0.046	**0.005**
SDy	TD	0.046 ± 0.045	-
	ADHD	0.088 ± 0.063	**0.016**
	drug-naïve ADHD	0.091 ± 0.049	**0.013**

Comparison of the mean standard deviation of horizontal (SDx) and vertical (SDy) gaze positions across the three participant groups. The p-values, derived from the Mann-Whitney U test, represent the statistical comparison of each ADHD group against the TD group. Significant values ($p < 0.05$) are highlighted in bold.
