# Supplementary material for: Multimodal biomarker based on temporal complexity of eye movements and pupil diameter in attention-deficit/hyperactivity disorder
Source: PLOS Ment Health. 2025 Oct 9;2(10):e0000456. doi: 10.1371/journal.pmen.0000456 (PMC12798525; doi:10.1371/journal.pmen.0000456)
Supplement: S2 Text — (PDF) [file pmen.0000456.s005.pdf]

S2 Text. Sensitivity Analysis

A sensitivity analysis was conducted to confirm that the classification results of this study are robust against potential low-frequency noise derived from head motion. The primary analysis, reported in the main text, used a 0.1-30 Hz band-pass filter to retain physiological eye-movement signals while suppressing noise. To test for any residual bias from head motion, which is known to occur primarily below 2 Hz [1, 2], the entire analysis pipeline was re-run using more aggressive high-pass filter settings (2-30 Hz and 4-30 Hz). The results of the sensitivity analysis show that the performance of the classifier was very stable across all filter settings for both the TD vs ADHD and TD vs drug-naïve ADHD comparisons (S2A Table). Notably, the model combining pupil diameter and a single eye-movement complexity (FuzzyEn) feature—which achieved the highest accuracy in the primary analysis—consistently maintained superior performance compared to all other feature combinations, regardless of the filter setting. This finding further reinforces the study's main conclusion that combining pupil-derived and eye-movement-derived features is the most effective classification strategy.

S2A Table. Sensitivity analysis of classification performance with different high-pass filter settings.

Model Features	0.1-30 Hz		2-30 Hz		4-30 Hz	
	AUC-ROC	AUC-PR	AUC-ROC	AUC-PR	AUC-ROC	AUC-PR
TD vs ADHD						
Pupil Size	0.76	0.65	0.76	0.65	0.76	0.65
Hor FuzzyEn	0.65	0.59	0.62	0.57	0.61	0.57
Vert FuzzyEn	0.72	0.71	0.64	0.60	0.68	0.63
Pupil Size + Hor FuzzyEn	0.79	0.73	**0.78**	0.72	0.78	0.68
Pupil Size + Vert FuzzyEn	**0.83**	**0.79**	0.77	**0.74**	**0.80**	**0.73**
Hor FuzzyEn + Vert FuzzyEn	0.72	0.70	0.62	0.61	0.66	0.62
Pupil Size + Hor FuzzyEn + Vert FuzzyEn	0.79	0.73	0.74	0.70	0.75	0.56
TD vs drug-naïve ADHD						
Pupil Size	0.77	0.65	0.77	0.65	0.77	0.65
Hor FuzzyEn	0.78	0.60	0.79	0.58	0.75	0.57
Vert FuzzyEn	0.75	0.67	0.70	0.60	0.75	0.63
Pupil Size + Hor FuzzyEn	**0.83**	0.68	**0.84**	0.69	**0.80**	0.68
Pupil Size + Vert FuzzyEn	0.82	**0.73**	0.81	**0.73**	**0.80**	**0.73**
Hor FuzzyEn + Vert FuzzyEn	0.75	0.57	0.75	0.56	0.75	0.62
Pupil Size + Hor FuzzyEn + Vert FuzzyEn	0.78	0.63	0.75	0.65	0.74	0.56

Comparison of model performance across all feature combinations and under three different high-pass filter settings (0.1-30 Hz, 2-30 Hz, and 4-30 Hz), using a Lasso logistic regression model. The analysis is separated by classification task. The highest value in each column for each task is highlighted in bold text. (TD, typical development; ADHD, attention-deficit/hyperactivity disorder; AUC-ROC, Area Under the Receiver Operating Characteristic Curve; AUC-PR, Area Under the Precision-Recall Curve)

References

[1] Keshner F, Peterson B. Mechanisms controlling human head stabilization. I. Head-neck dynamics during random rotations in the horizontal plane. Journal of neurophysiology. 1995;73(6):2293–2301.

[2] Stensdotter AK, DinhoffPedersen M, Meisingset I, Vasseljen O, Stavdahl Ø. Mechanisms controlling human head stabilization during random rotational perturbations in the horizontal plane revisited. Physiological reports. 2016;4(10):e12745.
