# Supplementary material for: Multimodal biomarker based on temporal complexity of eye movements and pupil diameter in attention-deficit/hyperactivity disorder
Source: PLOS Ment Health. 2025 Oct 9;2(10):e0000456. doi: 10.1371/journal.pmen.0000456 (PMC12798525; doi:10.1371/journal.pmen.0000456)
Supplement: S1 Fig — (PDF) [file pmen.0000456.s006.pdf]

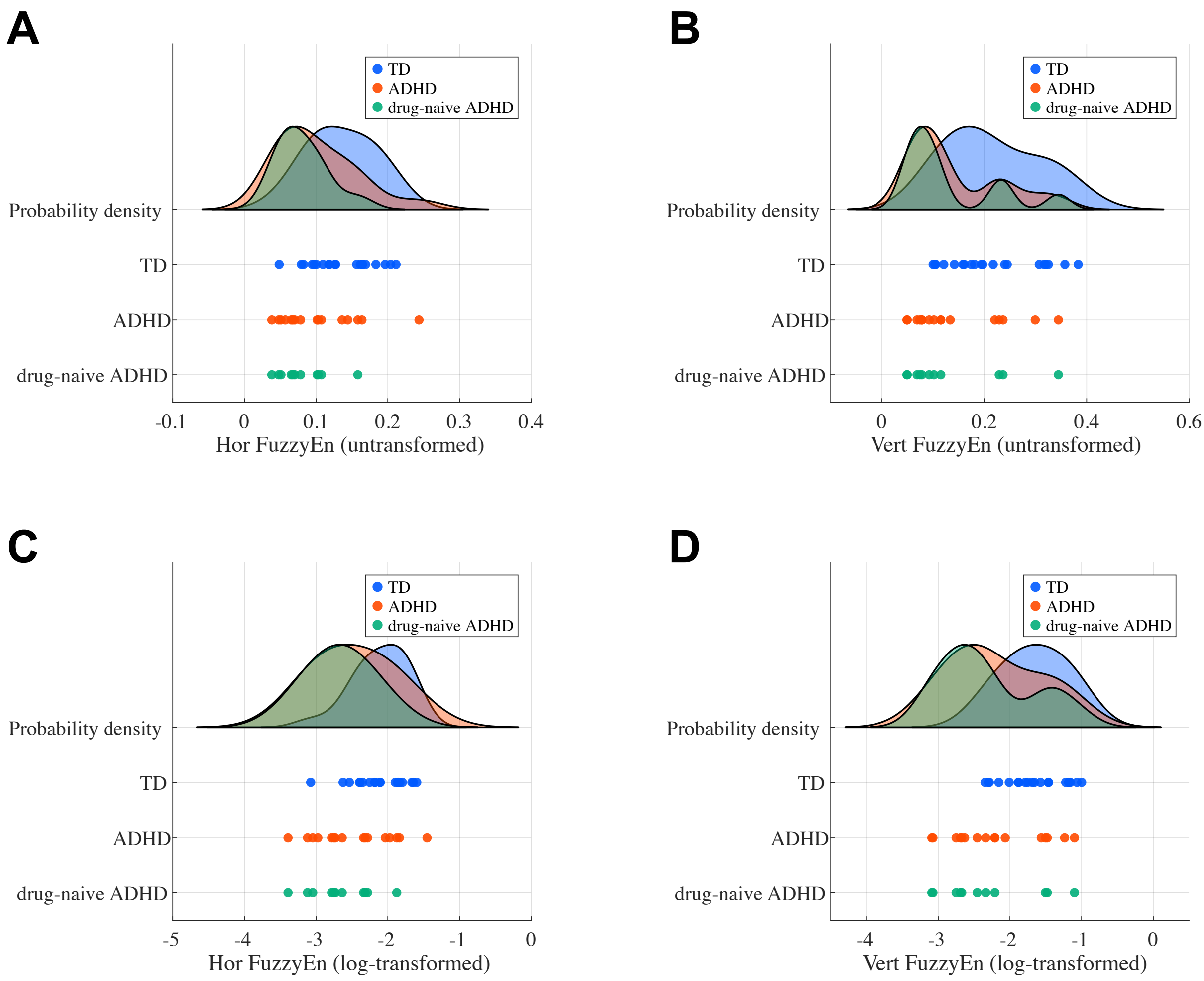

S1 Fig. Distributions of FuzzyEn features before and after log-transformation. Probability-density plots of FuzzyEn features, averaged across temporal scales 1–10, for three participant groups (TD, ADHD, and drug-naïve ADHD). Each panel shows overlaid kernel-density curves color-coded by group, with individual data points jittered along the x-axis for reference. (A, B) Untransformed horizontal and vertical FuzzyEn distributions, respectively. (C, D) Log-transformed horizontal and vertical FuzzyEn distributions, respectively. These log-scaled features—used in the classification analysis—are nearly symmetric and therefore more closely approximate a normal distribution.
