# Supplementary material for: Multimodal biomarker based on temporal complexity of eye movements and pupil diameter in attention-deficit/hyperactivity disorder
Source: PLOS Ment Health. 2025 Oct 9;2(10):e0000456. doi: 10.1371/journal.pmen.0000456 (PMC12798525; doi:10.1371/journal.pmen.0000456)
Supplement: Attachment — Submitted filename: Letter_Plos_Mental_Health.pdf [file pmen.0000456.s008.pdf]

[Date: August 15, 2025]

Dr. Zhiyi Chen
Academic Editor
PLOS Mental Health

We greatly appreciate the time and effort you and the reviewers dedicated to critically assessing our paper and are pleased to resubmit the revised version of our manuscript, "Multimodal biomarker based on temporal complexity of eye movements and pupil diameter in attention-deficit/hyperactivity disorder" (PMEN-D-25-00061), to *PLOS Mental Health*. Your comments, as well as those of the reviewers, were highly insightful and enabled us to improve the quality of our manuscript significantly. The manuscript has been revised following careful consideration of the reviewers' comments.

We look forward to working with you and the reviewers to move this manuscript closer to publication in PLOS Mental Health. Please find our detailed point-by-point responses below.

Editor comments (quoted):
"Thank you for the patience in waiting this decision. Though the Reviewer #2 recommend to accept this manuscript in the current form, I do reckon that the Reviewer #1 raised substantial solid concerns warranting an overhaul. I thus recommend a major revision before publication."
Response: Thank you for your feedback. We have revised the manuscript. We agree that Reviewer #1's concerns were substantial, and we have addressed all points through additional analyses and revisions to the text. Additionally, we have revised the manuscript, based on the Reviewer #2's comments. Please find our detailed point-by-point responses to the reviewers' comments below.

Reviewer Comments:

Reviewer #1 Comments:

We thank Reviewer #1 for their thorough and constructive feedback. We appreciate their assessment that our overall argument is plausible and well-supported by the literature. We have addressed each of the concerns as detailed below.

Major Concerns:

1. *The nature of the recorded (/alleged) eye movements*

- *How can the authors ensure that the measures fed into the analyses are indeed eye movements, and not head motion artifacts? Especially with ADHD, which is associated with the inability to stay still.*
- *Even with a chin rest, I doubt the lack of motion-induced noise in the data.*
- *To address this, either a thorough preprocessing removing artifacts is necessary, or at least the addition of a heatmap of the fixations to the paper. This additional figure could represent the single observations, or it could be the mean of each subgroup; the important thing would be to assess how big the eye movements are (because realistically, micro-saccades are associated with ADHD, not big eye movements).*

Response:

We thank the reviewer for highlighting the important issue of potential head motion artifacts. In response, we implemented a stepwise validation process, starting with spatial analyses, followed by more rigorous spectral and dynamical tests. This approach is summarized below and detailed in the revised manuscript (Discussion, para. 6) and the Supporting Information (S1 Text, S2 Text).

1. Initial spatial validation (heatmap and dispersion analysis):
   We investigated whether observed features could be explained by large head movements by generating heatmaps and quantifying standard deviations (SD) of horizontal and vertical gaze positions for each group using raw data (S1 Text; S1A Fig, S1A Table). Both ADHD and drug-naïve ADHD groups showed centrally clustered gaze but with significantly greater spatial dispersion than the TD group (all $p < 0.05$). This pattern aligns with literature describing increased fixational instability and higher frequency of microsaccades in ADHD (Armstrong & Munoz, 2003; Fried et al., 2014). However, as increased gaze dispersion could also result from head motion, we recognized this spatial analysis alone was insufficient to definitively rule

out motion artifacts.

2. Further spectral validation (sensitivity analysis):
   To examine head motion contamination, we used findings that involuntary head motion power is mostly below 2 Hz (Keshner & Peterson, 1995; Stensdotter et al., 2016), and physiological oculomotor signals (microsaccades, drift) occur mainly in the <10 Hz range (Mostofi et al., 2016). Thus, our analyses used a 0.1–30 Hz band-pass filter. We conducted a sensitivity analysis by re-running analyses with stricter high-pass filters (2 Hz and 4 Hz) to suppress low-frequency head motion. As described in S2 Text, classifier performance remained stable across all filter settings, with the model combining pupil diameter and a temporal complexity feature of eye movement consistently outperforming others. This indicates our results are robust against potential head motion artifacts.
3. Dynamic validation (surrogate data analysis):
   Finally, to determine whether the eye-movement signals reflected physiological oculomotor control rather than random or artifactual noise, we performed a surrogate data analysis (IAAFT). This revealed that the eye-movement time series exhibited nonlinear dynamics consistent with neural control mechanisms rather than stochastic motion noise (Fig 1B).

Following the reviewer's comments, we performed spatial analyses using gaze heatmaps and dispersion metrics and strengthened validation through additional spectral and dynamical analyses. These stepwise validation approaches strongly support our findings' robustness against head motion. Nevertheless, as noted in the Discussion, residual head movement effects cannot be fully excluded. Future studies using simultaneous eye and head movement tracking (e.g., accelerometers or motion-tracking systems) are needed to resolve this issue definitively.

2. *AUC may confound interpretation*
   - *AUC curves can often seem very good, but obscure the true classificatory prowess of models.*
   - *For example, a 2x2 confusion matrix showing the exact classifications could clarify the aptitude of the predictors and the models in general.*
   - *I strongly suggest the addition of a precision-recall curve to complement the AUC curves.*

Response:

We thank the reviewer for their insightful suggestions. We agree that relying solely on AUC-ROC can sometimes mask the weaknesses in a model's practical classification performance, particularly in the context of class imbalance. To address the potential limitations of the AUC-ROC, we revised our evaluation to include precision-recall (PR) curves and confusion matrices.

1. Addition of PR Curves and AUC-PR:
   Following the reviewer's recommendation, we supplemented our ROC analysis with PR curves and their corresponding Area Under the Curve (AUC-PR). All models were evaluated using this dual-metric approach, with the results presenting both AUC-ROC and AUC-PR values (Results, "Evaluation of classification ability using characteristics of eye movement and pupil diameter," paras. 2–3; Fig 3A and 3B).
2. Inclusion of 2×2 Confusion Matrices:
   To clarify the practical aptitude of our models, we provide 2×2 confusion matrices for the two best-performing classifiers (Fig 3C and 3D). The classification threshold for each matrix was systematically selected to maximize the F1-score on the corresponding PR curve. This methodology, along with key performance metrics such as sensitivity, specificity, and F1-score, is now detailed (Results, "Evaluation of classification ability using characteristics of eye movement and pupil diameter," para. 4).
3. Revised Interpretation of Results:
   We now explicitly state that the combined-feature model (Pupil Size + temporal complexity of eye movement) outperforms single-feature models across both AUC-ROC and AUC-PR metrics, reinforcing our central conclusion (Results, "Evaluation of classification ability using characteristics of eye movement and pupil diameter," paras. 2–3). This revised interpretation demonstrates, as the reviewer noted, that the PR curve provides a more nuanced assessment of performance. We also maintain our emphasis that these findings are preliminary and necessitate external validation.

Collectively, these revisions offer a more comprehensive and multifaceted evaluation of the classification power of our model, directly addressing the reviewer's valuable feedback. We are confident that these enhancements significantly strengthen the manuscript.

3. Logistic models

- *Logistic regressions often overfit models with such small sample sizes, even with*

cross-validation. Was any alternative method considered?

- *It is good that ROC curves and cross-validation accuracy were reported, but it should be acknowledged in the limitations paragraph of the discussion.*
- *Additionally, the unbalanced group sizes can skew results and should also be acknowledged.*
- *Although the small sample size was addressed, the implications of this weren't and can result in misinterpretation on the generalizability and veracity of the findings.*

Response:

We thank the reviewer for underscoring the dangers of overfitting and class imbalance in small-sample studies. In our revised manuscript, we have taken several concrete steps to address these valid concerns and substantially expanded the discussion of the attendant limitations.

1. Primary Classifier:
   In response to the reviewer's concern about overfitting, we revised our model to use L1-regularized (Lasso) logistic regression (Tibshirani, 1996), which performs simultaneous feature selection and shrinkage to prevent overfitting in small datasets (Methods, "Machine learning for classification," paras. 1–3).
2. Robustness Checks:
   To confirm our findings' stability, we refitted the data with two additional algorithms, standard logistic regression and Firth's bias-reduced logistic regression (Firth, 1993), under identical cross-validation and weighting schemes. The results in the new S3 Table show that the model combining pupil size and temporal complexity of eye movement remains the top performer across classifiers. (Methods, "Machine learning for classification," paras. 1–4; S3 Table).
3. Class-imbalance Control:
   We implemented a weighting scheme with observations weighted inversely by class frequency, a standard technique for addressing class imbalance (He & Garcia, 2009). This approach prevents model bias toward the majority TD group (Methods, "Machine learning for classification," para. 2).
4. Expanded Limitations Section:
   The Discussion's opening paragraph now identifies "small sample size and moderate class imbalance" as the study's principal limitations. We elaborate on their impact on generalizability, model stability, and overfitting. We now state that classification metrics should be considered preliminary and emphasize that validation in larger, independent cohorts is necessary to

establish these findings' generalizability (Discussion, para. 5).

We believe that these revisions directly and comprehensively address the reviewer’s concerns while keeping the manuscript concise and transparent.

Minor Concerns:

1. *The authors should discuss or hypothesize why the measures do not correlate with ADHD severity, given that the measures are supposed to serve as an ADHD biomarker. This finding is counter-intuitive and should thus be addressed. Discuss what the utility of a biomarker is that does not differ between disorder severity?*

Response:

We thank the reviewer for highlighting this important finding. We agree that a biomarker's utility without symptom severity correlation warrants careful consideration. In response, we added a paragraph in the Discussion to address this and hypothesize underlying reasons (Discussion, para. 8). Our revised manuscript response includes three key points.

1. Statistical Power:
   We acknowledge our analysis may have been underpowered to detect a modest correlation, given the limited sample size (16 ADHD or 11 drug-naïve ADHD participants). Power analysis suggests a larger cohort (~60 cases) would be required to confirm such a relationship with sufficient statistical confidence.
2. Potential for Nonlinear Relationships
   We further hypothesize that the relationship between neurophysiological measures and ADHD symptom severity may be non-linear. For example, the inverted-U relationship between LC-NE system arousal and cognitive performance (Aston-Jones & Cohen, 2005; Lu et al., 2023), which is indexed by pupil diameter, could obscure a linear association. A similar effect may apply to temporal complexity of eye movements.
3. Utility of Categorical Biomarkers
   We emphasize that many clinically established biomarkers provide substantial diagnostic or prognostic value without strong associations with disorder severity. For example, the SARS-CoV-2 cycle threshold (Ct) value and amyloid-PET imaging for Alzheimer's disease offer robust categorical information for diagnosis or risk stratification, despite modest correlations with clinical severity (Shah et al., 2021; Mosconi & McHugh, 2011). Our findings suggest these biomarkers' utility may lie in supporting categorical diagnosis

or screening rather than tracking symptom progression.

We are grateful for the opportunity to clarify these important issues. We believe that the revised discussion now provides a more nuanced interpretation of our findings and the potential clinical roles of these measures.

2. *Histograms of the main measures/predictors (before log-transformations) would be helpful to see if the distributions overlap.*

Response:

We appreciate this suggestion. To visualize group-wise distributions of main predictors and clarify the rationale for log-transformation, we have added S1 Fig, showing probability-density plots (kernel-density estimates with overlaid data points) for scale-averaged (temporal scales 1–10) horizontal and vertical FuzzyEn features, before and after log transformation. We chose density plots over binned histograms as they convey the same information while avoiding arbitrary bin-width choices. As S1 Fig shows, untransformed features are skewed, whereas log-transformed features are nearly symmetric and better separated, confirming the appropriateness of this preprocessing step. The figure is now cited in the main text (Results, "Analysis of eye movement dynamics," para. 2; Methods, "Statistical analysis," para. 1). These additions fully address the reviewer's comment and enhance analysis transparency.

3. *Clarity of concepts*

- *Some concepts and disorders could have been briefly described, since some are not commonly known of, e.g., "dysmetria". A short sentence like "with and without dysmetria, a neurological disorder affecting fine motor skills..." would already suffice.*
- *Similarly, defining the meaning of eye movement complexity in the beginning would facilitate understanding, as the term is used for the general concept of eye movement, and for the time-adapted operationalization in this paper, i.e., temporal complexity. It is often diffuse how eye movement complexity is operationalized through MSE as FuzzyEn, and when the general term is meant, or the temporal concept.*
- *Similarly, "baseline pupil diameter" suggests that pupil diameter was only assessed at the beginning of the measurement, instead of the time-averaged measure that it is. Perhaps rephrasing "baseline pupil diameter" to "mean pupil diameter during the complete fixation period" clarifies this.*

Response:

Thank you for your insightful and constructive suggestions. We agree that your suggestions have significantly improved the clarity and the readability of our manuscript. We have addressed each of these points in detail below.

1. Definition of “dysmetria”:

   As suggested, we have now added a brief definition of “dysmetria” to clarify this term for the reader (Introduction, para. 3). The revised sentence now reads: “Furthermore, a study on patients with cerebellar ataxia showed significant differences in the temporal complexity of eye movements between patients with and without dysmetria (a condition characterized by impaired movement coordination, often resulting in an undershoot or overshoot of the intended target).”
2. Definition of "eye movement complexity”:

   We agree that our initial use of "eye movement complexity" could be ambiguous, mixing the general concept with the specific "temporal complexity" used in our study. To address this, we have revised the manuscript to consistently use "temporal complexity" when referring to the metric evaluated by our entropy analysis. We have now defined the term (Introduction, para. 2): "temporal complexity defined as the degree of irregularity or unpredictability of time-series patterns is quantified using entropy-based indices such as sample entropy and fuzzy entropy (FuzzyEn); higher values indicate greater irregularity."
3. Rephrasing of “baseline pupil diameter”

   We agree that "baseline pupil diameter" could be misleading. We have replaced this term with "mean pupil diameter" throughout the manuscript and added a clear operational definition to the Methods section ("Analysis of mean pupil diameter"). The Methods section now defines this metric: "To quantify each participant's overall pupil size, we calculated the "mean pupil diameter," defined as the temporal average of pupil diameter across all valid epochs of the complete fixation period. These values were averaged between the right and left eyes."

4. *Measurement conditions*

- *The experimental conditions are not fully described in my opinion.*
- *The utilization of mean pupil diameter seems in agreement with literature, but diameter is heavily influenced by other factors. For example, was every participant measured at the same time of the day, since fatigue plays a role in pupil size? Even*

eye color can play a role, since, e.g., with the same amount of light, darker eyes tend to have less dilated pupils than lighter colored eyes. Was any of this controlled for? If so, it should be described in the section on measurement.

Response:

Thank you for your valuable and constructive feedback on our manuscript. We agree that clarifying the experimental conditions is crucial for ensuring the reliability of our findings. We have revised the manuscript to address your specific concerns regarding the factors that could influence pupil diameter.

1. Regarding the time of day (fatigue):
   We agree with the reviewer that factors like fatigue can affect pupil size. To address this, we recorded each participant's measurement time and compared groups. The mean start time (minutes from midnight) for all groups was in the early afternoon (TD = 874 ±136 min, ADHD = 862 ± 99 min, drug-naïve ADHD = 846 ± 91 min; ≈ 14:00-14:35), and neither comparison (TD ADHD, TD drug-naïve ADHD) was statistically significant ($p > 0.55$; Table 2), minimizing the impact of time of day or fatigue on results (Methods, “Recording of eye movements and pupil diameter,” para. 1; Table 2).
2. Regarding Eye Color:
   We agree that iris pigmentation acts as a confounding variable. To minimize this, we recruited only Japanese participants, an ethnic group with virtually universal dark-brown irises (Iida et al. 2009; Murray et al. 2015). Consequently, the sample was homogeneous regarding iris color (Methods, “Participants,” para. 2). Additionally, all measurements were taken under identical photopic lighting in a lit room (Methods, “Recording of eye movements and pupil diameter,” para. 1), further reducing any residual variance related to pigmentation.

5. *Main measures: Pupil size*

- *Was it considered to assess pupil size variability, not just pupil size mean? Variability may have more classification power than just utilizing the mean, indicated by the statement in lines 44 to 46 (“the temporal complexity of pupil diameter is important for capturing the pathophysiology of ADHD, and its relationship with the LC-NE system has been reported”).*
- *Perhaps an analysis including pupil variability or some kind of time variance data on pupil size can be added.*

Response:

We thank the reviewer for this insight. As noted in Introduction, pupil diameter variability is an important indicator of ADHD pathophysiology (Nobukawa et al., 2021; Kumano et al., 2022). The reviewer's question about mean pupil diameter for our analysis is relevant. Our primary aim was to determine the additional diagnostic value of temporal complexity feature of eye movement when combined with a well-established pupillary marker. To isolate the contribution of this new feature and mitigate overfitting with our limited sample size—a valid concern raised by the reviewer—we chose mean pupil diameter. This decision was based on its utility as a foundational metric, supported by findings that adults with ADHD exhibit larger baseline pupil diameters (Shirama et al., 2020; Nobukawa et al., 2021). This choice is a limitation, as variability-based metrics likely contain complementary information not captured by the mean alone (Nobukawa et al., 2021). Future work should leverage larger datasets to develop sophisticated classifiers that integrate the complexity of both eye movements and pupil diameter, which may further improve diagnostic accuracy (Discussion, para. 7).

6. *Multicollinearity*

- *How was the threshold for Pearson's r to assess multicollinearity established? Are there guidelines for this?*
- *Why weren't other multicollinearity measures used instead, e.g., VIF or VDPs?*
- *Multicollinearity could've been tested in the models themselves, without having to remove multicollinear effects a priori.*

Response:

Thank you for your constructive comments regarding multicollinearity. Your feedback prompted us to revise and strengthen our analytical approach.

1. Revised Approach to Multicollinearity
   We agree that multicollinearity should be addressed within the model. Following your suggestion, we changed our primary classifier to an L1-regularized (Lasso) logistic regression model (Tibshirani, 1996) (Methods, "Machine learning for classification"). This method manages multicollinearity by performing feature selection and shrinking coefficients of redundant predictors. By managing multicollinearity within the model, this approach eliminates the need for VIF or correlation features. The correlation analysis now serves as preliminary evidence of collinearity and reinforces the need for Lasso (Results, "Evaluation of classification ability using characteristics of eye movement and pupil diameter," para. 1; S1 Table).

2. Empirical Validation of Approach:
   To validate our approach, we systematically evaluated all feature combinations (S2 Table). This analysis confirmed that models with redundant features performed poorly, while the most parsimonious model combining Pupil Size and a single eye-movement feature achieved the best performance (Results, "Evaluation of classification ability using characteristics of eye movement and pupil diameter," para. 2; Discussion, para. 5).

In conclusion, your feedback was instrumental in strengthening our analyses. By adopting L1 regularization, we addressed multicollinearity within the model, a choice validated by our systematic performance evaluation.

7. *Was the epoch rejection/inclusion rate similar across the three subgroups, since noisy epochs were removed? This could also influence results.*

Response:

Thank you for your insightful comments. We agree that this is a crucial point for ensuring the reliability of our results. As suggested, we calculated and compared the epoch rejection rates across the three subgroups (TD, ADHD, and drug-naïve ADHD). The results showed no statistically significant difference in rejection rates between the TD (12.1%(mean) ± 27.9%(SD)), ADHD (20.3%(mean) ± 29.2%(SD)), and drug-naïve ADHD groups (22.7%(mean) ± 32.9%(SD)) (TD vs ADHD, $p = 0.395$; TD vs drug-naïve ADHD, $p = 0.348$). This finding suggests that the removal of noisy epochs was not biased across the groups and that data quantity is unlikely to be a confounding factor in our results (Methods, “Recording of eye movements and pupil diameter,” para. 2).

8. *Interpretability of the MSE results*

- *Complexity differences might indeed reflect neurophysiological alterations or be mere noise. A further control analysis, for example using completely shuffled time series, could confirm the findings and substantiate that entropy differences are not driven by measurement artifacts.*

Response:

We thank the reviewer for this suggestion. To verify our results reflect genuine neurophysiological dynamics rather than measurement artifacts, we performed analysis. We generated surrogates using the Iterative Amplitude-Adjusted Fourier Transform (IAAFT) method (Schreiber & Schmitz, 1996), a more rigorous control

than random shuffling (Theiler et al., 1992). While random shuffling destroys temporal structure, IAAFT preserves the linear properties of the original signal—its power spectrum and amplitude distribution—while randomizing Fourier phases to eliminate nonlinear temporal structure. The results (Fig 1B) show that FuzzyEn values of IAAFT surrogates were significantly higher than those of the original time series, allowing us to reject the null hypothesis that the data arise from a purely linear stochastic process. This confirms the reported complexity differences reflect nonlinear neural dynamics (Discussion, para. 2). Details of this validation have been added to the manuscript (Methods, "Surrogate data analysis").

Reviewer #2 Comments:

We thank Reviewer #2 for their constructive evaluation, particularly for highlighting the clinical relevance of our findings and technical rigor. The reviewer raised two key challenges we have addressed to strengthen the case for future clinical translation: first, the limited sample size and second, the need for external validation. Below, we respond to each point and detail the revisions made to the manuscript.

1. *Limited sample size*

Reviewer comment (paraphrased): The findings are compelling, but the sample size is limited.

Response:

We are grateful to the reviewer for raising this crucial point. We agree and have explicitly acknowledged the relatively small sample size as a primary limitation of our study in the revised manuscript. We elaborate on how this impacts the generalizability of our findings and the reliability of our classification model. Furthermore, we also discuss how the limited statistical power due to the small sample size may explain the lack of correlation between our biomarkers and symptom severity (Discussion, paras. 5, 7).

2. *Need for external validation*

Reviewer comment (paraphrased): External validation is needed before clinical application.

Response:

We agree with the reviewer's assessment. The establishment of clinical utility requires rigorous external validation, and we have underscored this in the revised Discussion. We state that the reliability and clinical utility of our model must be established through validation on larger independent datasets. Our study provides a foundation for such future large-scale validation studies, which are essential for clinical translation. (Discussion, para. 5).

We thank you and the reviewers for your valuable comments. We believe the revised version is suitable for publication in *PLOS Mental Health*.

Sincerely,

Sou Nobukawa, PhD,
Professor
Department of Computer Science, Chiba Institute of Technology, Narashino, Chiba, Japan
Email: nobukawa@cs.it-chiba.ac.jp
